# Personalizing IL-23 Inhibitor Therapy in IBD: Current Evidence and Future Directions in Therapeutic Drug Monitoring and Dose Optimization

**DOI:** 10.3390/jcm14217471

**Published:** 2025-10-22

**Authors:** Alessandro Pedicelli, Talat Bessissow, Waqqas Afif

**Affiliations:** Department of Medicine, Division of Gastroenterology & Hepatology, McGill University Health Centre, 1001 Decarie Blvd, Montreal, QC H4A 3J1, Canada

**Keywords:** inflammatory bowel disease, therapeutic drug monitoring, IL-23 inhibitor

## Abstract

Interleukin-23 (IL-23) inhibitors have rapidly become an essential component of the therapeutic armamentarium for inflammatory bowel disease (IBD). Risankizumab, mirikizumab, and guselkumab share broadly similar pharmacokinetic and pharmacodynamic properties, including linear clearance, long half-lives, and low immunogenicity. While therapeutic drug monitoring (TDM) is well established in the use of anti-TNF agents, its role in IL-23 inhibitors remains undefined. Emerging evidence, mostly for risankizumab, demonstrates dose–response relationships, suggests potential maintenance thresholds, and outlines possible dose optimization strategies. However, this preliminary data is predominantly retrospective, often single-center, and involves a small number of patients. Until more robust evidence supporting the efficacy of TDM and dose optimization emerges, routine use of this clinical practice in IBD remains investigational.

## 1. Introduction

Inflammatory bowel diseases (IBD), which include Crohn’s disease (CD) and ulcerative colitis (UC), are immune-mediated disorders that primarily affect the gastrointestinal tract. Patients diagnosed with IBD often require life-long therapy to reduce the risk of hospitalization and surgery as well as improve quality of life [1]. In Canada, like many other parts of the world, the prevalence of IBD is increasing, with more than 1% of the general population expected to have the diagnosis by the year 2035 [2]. Over the past two decades, a growing armamentarium of advanced therapies have proven to be safe and effective in treating both CD and UC [1,3]. Currently available treatment options include drugs that target IL-23, IL-12/IL23, JAK, sphingosine-1-phosphate, integrins, and tumor necrosis factor (TNF) alpha [4]. With the advent of expanded therapeutic options, there has also been a focus on maximizing therapeutic effect, while minimizing loss of response and safety concerns. Therapeutic drug monitoring (TDM)—which involves measuring drug serum trough levels and anti-drug antibodies—has emerged as a useful tool to optimize advanced therapies. The evidence is especially robust in the case of anti-TNF agents (i.e., infliximab and adalimumab), where reactive TDM and dose optimization have been shown to improve clinical and biochemical remission rates in patients with CD and UC [5]. The role of TDM with non-TNF-targeting agents is less well established, with no clear guidelines pertaining to proactive or reactive TDM [3].

Under homeostatic conditions, IL-23 is expressed by dendritic cells and macrophages throughout the normal bowel, particularly in the terminal ileum, as a reaction to microbial stimulation. The role of IL-23 in normal physiology is to promote the induction and differentiation of Th17-differentiated CD4+ T cells, which in turn release several cytokines including IL-17, IL-22, and interferon gamma. The downstream effects of these cytokines are meant to promote intestinal barrier integrity; however, their effects can quickly become pathogenic should the expression of IL-23 become excessive [1]. Animal studies and models have consistently shown that elevated mucosal concentration and expression of IL-23 are associated with intestinal inflammation and colitis. Conversely, knockout mice with no IL-23p19 subunit expression are protected against the development of colitis. Additionally, in humans, genome-wide association studies have shown that patients with loss-of-function polymorphisms of genes involved in the IL-23 pathway are significantly less susceptible to developing IBD over their lifetime [1]. Taken together, these data elucidate the pathological role of IL-23 in driving intestinal inflammation, thus making it an attractive target to treat IBD.

Within the class of IL-23 inhibitors, three molecules are currently approved for the treatment of moderate-severe CD and UC: risankizumab (RZB), mirikizumab (MIRI), and guselkumab (GUS). These agents target the p19 subunit of IL-23, which inhibits its interaction with the IL-23 receptor, leading to reduced downstream signaling and gene transcription of proinflammatory cytokines [1,4]. Pivotal trials have shown these drugs to increase clinical, biochemical, and endoscopic remission rates in IBD patients when compared to placebo and, in certain cases, Ustekinumab [4,6]. However, a proportion of anti-IL-23 treated patients still experience inadequate or loss of response to these advanced therapies, thus prompting the question of whether or not TDM or dose optimization strategies can recapture efficacy. There is a paucity of studies relating to the potential clinical utility of TDM and dose optimization of the IL-23 inhibitors. In this narrative review, we summarize the pharmacokinetic (PK) and pharmacodynamic (PD) properties of these medications as well as examine the evidence pertaining to TDM and dose optimization of IL-23 inhibitors.

## 2. Risankizumab

### 2.1. Pharmacokinetics and Pharmacodynamics

Risankizumab was the first IL-23p19 inhibitor to be approved for IBD, coming to market in 2022 as a treatment for Crohn’s disease. It exhibits linear pharmacokinetics with dose-proportionate increases in serum levels being observed with repeated drug administration [3,7]. Steady-state concentrations are achieved 16 weeks after the standard induction regimens and the drug exhibits a half-life of 21–28 days in both CD and UC [3]. Like all monoclonal antibodies, RZB is degraded via intracellular catabolic pathways into smaller peptides and amino acids. As such, no hepatic or renal clearance is observed and no dose adjustments are required for patients with chronic kidney or liver disease [1]. In Crohn’s disease, baseline body weight, sex, albumin, C-reactive protein (CRP), serum creatinine, and corticosteroid use have been shown to affect drug clearance, though these factors do not significantly alter clinical drug efficacy or safety [7]. In ulcerative colitis, high baseline body weight and low albumin have been shown to increase drug clearance, though once again, the magnitude of these effects was not clinically relevant [8].

The induction dose of RZB differs between CD and UC. In Crohn’s disease, induction with 600 mg IV at weeks 0, 4, and 8, achieves the plateau of the dose–response curve, thus exhibiting near-maximal clinical efficacy [7]. Larger induction doses, like the 1200 mg regimen investigated in the phase 3 ADVANCE and MOTIVATE trials, confer no additional clinical efficacy [6,7,9]. In UC, induction is achieved with 1200 mg IV at weeks 0, 4, and 8, which leads to near maximal efficacy across all clinical, biochemical, and endoscopic endpoints. Induction doses of 600 mg proved suboptimal, while an 1800 mg regimen provided little added benefit [8].

When it comes to maintenance, RZB is available at doses of 180 mg or 360 mg delivered subcutaneously (SC) every 8 weeks for both UC and CD. Population PK/PD analyses from the pivotal CD trials demonstrate higher 52-week endoscopic remission rates with the 360 mg regimen as compared to the 180 mg maintenance group, with only a minimal difference observed in clinical endpoints [7]. In UC, comparable PK/PD analyses of the landmark trials revealed numerically higher efficacy rates in 360 mg maintenance group at week 52 versus the lower dosing regimen. However, these rates were not statistically significant with a large overlap of the resulting confidence intervals, suggesting that there is no clinically relevant difference between the maintenance doses. This equivalency remained when analyzing subgroups stratified by baseline FCP, CRP, sex, advanced therapy exposure, and disease extent [8].

No dose-related safety signals were identified within any of the induction or maintenance regimens across both Crohn’s and ulcerative colitis [7,8]. In terms of immunogenicity, the development anti-drug antibodies (ADA) against RZB is rare, occurring in only 1–2% of patients in the landmark phase 3 trials [4]. Most of these ADA were non-neutralizing and did not impact drug exposure, clinical efficacy, or endoscopic response [3,4].

### 2.2. Therapeutic Drug Monitoring and Dose Optimization

Despite the PK and PD of risankizumab exhibiting clear dose–response properties, there remains a paucity of published data exploring the relationship between serum drug levels and clinical, biochemical, and endoscopic outcomes. A single-center prospective study of 28 patients with CD conducted by Roblin et al. [10] showed that mean maintenance trough concentrations of RZB were significantly higher in patients in clinical and biochemical remission as compared to those not in remission (21.6 ± 13.3 vs. 7.4 ± 6.4 μg/mL; *p* = 0.001) at 18 months. Additionally, there was a dose–response effect, with increasing RZB trough concentrations being associated with higher rates of remission. Receiver operating characteristic (ROC) curve analysis (area under the ROC curve [AUC], 0.93; *p* < 0.001) identified a maintenance trough level above 11.5 μg/mL to be significantly associated with clinical and biochemical remission (sensitivity 81.8%, specificity 80.3%) [10]. This same group had previously shown that serum RZB levels as early as week 4 are significantly higher in patients in biochemical remission six months later during maintenance treatment, thus suggesting a predictive value of early RZB trough levels [11]. Taken together, this data introduces the scientific plausibility and possible clinical utility of TDM in risankizumab treatment.

The question of dose optimization as a means to recapture patients experiencing inadequate response to RZB was investigated by Baert et al. [12] who analyzed subgroup data from the FORTIFY trial, a phase 3, double-blind, re-randomized responder withdrawal, maintenance study. In it, patients that had initially responded to the standard 12-week IV induction were re-randomized to RZB 180 mg SC, RZB 360 mg SC, or placebo every 8 weeks for 52 weeks. Starting at week 16, patients with inadequate or loss of response, were eligible to receive a rescue therapy (one dose of RZB 1200 mg IV, followed by RZB 360 mg SC every 8 weeks). Results from this trial showed that administration of the rescue regimen was able to re-capture clinical remission and/or endoscopic response in 20–36% of those patients experiencing inadequate response to RZB [12]. Furthermore, in a retrospective analysis of 20 patients with CD or UC and inadequate response to RZB maintenance therapy, Schreiber-Stainthorp et al. [13] showed that dose escalation to 360 mg SC every 4, 6, or 7 weeks led to a subjective improvement in clinical symptoms in 70% of patients as well as significantly increased hemoglobin, albumin, and body weight as compared to baseline values. Similarly, a 2024 retrospective cohort analysis by Nichols et al. [14] showed that dose escalation of RZB to every 4 or 6 weeks in patients with clinically or endoscopically active CD led to symptomatic improvement in 50% (*n* = 6/12) of study participants.

Finally, in a cohort study of 17 CD patients with loss of or inadequate response to RZB, Dalal et al. [15] reported that, in patients who underwent dose intensification to 360 mg SC every 4 or 6 weeks, 71% achieved clinical response, 53% achieved steroid-free clinical remission, and 67% had endoscopic improvement on follow-up colonoscopy [15].

Collectively, these studies provide evidence supporting the potential clinical utility of therapeutic drug monitoring and dose optimization of risankizumab by elucidating a potential target trough level, delineating dosing escalation regimens, and offering early clinical and endoscopic efficacy correlates.

## 3. Mirikizumab

### 3.1. Pharmacokinetics and Pharmacodynamics

Mirikizumab is a humanized IgG4 monoclonal antibody targeting the IL-23p19 subunit that was first approved for ulcerative colitis in 2023. Pharmacokinetic analyses from phase II and III UC trials demonstrated that MIRI exhibits linear pharmacokinetics, a terminal half-life of approximately 9.5 days, and is degraded by intracellular proteolysis, thus negating the effects of renal and hepatic function on drug clearance [16,17]. Covariate analyses have shown that body weight, body mass index, and albumin exert statistically significant effects on clearance and distribution. However, the magnitude of these effects is small, clinically irrelevant, and does not warrant dose adjustment in practice [16].

Phase II dose-finding trials have determined that the standard induction and maintenance regimens (CD: 900 mg IV at weeks 0, 4, 8 followed by 300 mg SC q4w; UC: 300 mg IV at weeks 0, 4, 8 followed by 200 mg SC q4w) achieve near maximal efficacy, with larger doses providing minimal further benefit [4,17]. In the pivotal LUCENT trials, 23% of MIRI-treated patients developed anti-drug antibodies, but only 2.6% of patients exhibited reduced drug levels, leading to negligible clinical effects [4].

### 3.2. Therapeutic Drug Monitoring and Dose Optimization

Unlike risankizumab, there are currently no proposed serum trough thresholds for mirikizumab in IBD. Although higher serum drug levels initially appeared to be associated with greater rates of clinical, endoscopic, and histologic remission, these relationships weakened significantly when adjusted for confounding [17]. Thus, the current data do not support proactive TDM.

With respect to dose optimization, a continuation study conducted by Sandborn et al. [18] showed that among UC patients with an inadequate response to standard induction, extending the induction period with larger doses (600 or 1000 mg IV q4weeks) for an additional 12 weeks led to a clinical response in up to 50% of patients. As such, extended induction or re-induction of MIRI may be a potential salvage strategy for patients with partial or loss of response. However, real-world data supporting these regimens is lacking.

## 4. Guselkumab

### 4.1. Pharmacokinetics and Pharmacodynamics

Guselkumab is a fully human IgG1λ monoclonal antibody against IL-23p19. While only recently approved for UC and CD, GUS has been available as a treatment for plaque psoriasis since 2017 and psoriatic arthritis since 2020 [19]. A recent analysis of the phase II and III induction and phase III maintenance trials of GUS in UC determined that the drug exhibits linear pharmacokinetics with a terminal half-life of approximately 16.5 days. Covariate analysis identified body weight, serum albumin, CRP, age, sex, and prior biologic failure status as statistically significant factors in drug clearance. However, none produced clinically meaningful effects, thus negating the need for dose adjustment [20]. The IV induction dose of 200 mg at weeks 0, 4, and 8 appears to achieve near-maximal efficacy, with larger doses garnering no additional benefit in the Phase 2 GALAXI-1 and QUASAR trials [21,22]. Unique to GUS in CD, induction with 400 mg SC q4w for three doses has been shown to significantly improve clinical and endoscopic outcomes at week 12 when compared to placebo [23]. Preliminary evidence supporting SC induction in UC has also recently been presented, but this regimen has yet to be approved for clinical use [24]. Maintenance doses of 100 mg SC every 8 weeks or 200 mg SC every 4 weeks are available for both CD and UC. While the larger 200 mg dose exhibited numerically higher efficacy in the pivotal maintenance trials, these differences were not statistically significant and are unlikely to be clinically relevant [25]. Immunogenicity data in the IBD population are not yet available, but studies from dermatology and rheumatology indicate a low incidence of anti-GUS antibodies, typically non-neutralizing and without meaningful impact on PK or efficacy [19]. This profile likely carries over to UC and CD.

### 4.2. Therapeutic Drug Monitoring and Dose Optimization

Unlike risankizumab, there are no IBD-specific TDM data for guselkumab. The current dosing regimens already appear to achieve maximal efficacy, and no exposure–response targets have been defined [1]. As such, therapeutic drug monitoring has no current role in guselkumab therapy. Similarly, dose optimization strategies (such as interval shortening or re-induction) have not been systematically evaluated and are not part of standard practice.

## 5. Conclusions

IL-23p19 inhibitors represent a major advance in the management of inflammatory bowel disease. Among them, risankizumab, mirikizumab, and guselkumab share broadly similar pharmacokinetic and pharmacodynamic characteristics, with linear clearance, long half-lives, and minimal clinically relevant effects of covariates such as weight, albumin, or baseline inflammatory status. Immunogenicity rates are consistently low across the class, with most anti-drug antibodies being non-neutralizing and without meaningful clinical impact.

Of the three agents, risankizumab currently has the most robust PK/PD data in IBD. Dose–response relationships are well established, and emerging evidence supports the potential clinical utility of therapeutic drug monitoring with a maintenance trough target of 11.5 μg/mL. Early trough concentrations appear predictive of subsequent outcomes, and dose optimization strategies have shown promise in patients with loss of response. However, the bulk of existing data comes from small, single-center, retrospective analyses, which limits validity and generalizability. Despite the commercial availability of RZB TDM testing [26], there exists a significant amount of uncertainty surrounding this clinical practice and the routine use of TDM and dose optimization remains premature. Additionally, given the low rates of immunogenicity of IL-23 inhibitors, the utility of proactive TDM during maintenance therapy remains especially nebulous. While we do see a potential role for dose optimization in patients experiencing loss of or inadequate response to RZB, exact drug targets and other TDM parameters must still be elucidated.

Mirikizumab similarly demonstrates predictable PK and low immunogenicity, but exposure–response analyses are less clear-cut and real-world data is limited. Despite having some data in the rheumatology and dermatology literature, guselkumab remains largely uncharacterized in context of IBD-specific TDM and dose optimization. The findings pertaining to standard dosing regimens, potential TDM targets, and investigational optimization regimens are summarized in Table 1.

## 6. Future Directions

Despite encouraging findings, significant gaps remain in our understanding of therapeutic drug monitoring and dose optimization of IL-23 inhibitors in IBD. First, larger, prospective, multicenter studies are needed to validate target trough concentrations as predictors of remission. Such work should extend beyond clinical and biochemical endpoints to include validated endoscopic and histologic outcomes.

Second, the relationship between immunogenicity and clinical response warrants further exploration. Although ADA formation is rare, larger real-world registries may be required to clarify whether low-level or transient antibodies have subtle effects on disease outcomes over longer follow-up.

Finally, dose optimization strategies require more rigorous evaluation. While interval shortening and IV re-induction appear effective in subsets of RZB-treated patients, these approaches remain supported mainly by retrospective or post hoc analyses. Prospective, randomized trials examining proactive versus reactive optimization strategies are needed to establish standard-of-care algorithms.

## Figures and Tables

**Table 1:** Summary of standard dosing regimens, potential therapeutic drug monitoring targets, and investigational optimization strategies.

	Induction	Maintenance	Target Trough (μg/mL)	Optimization Strategy
Risankizumab	CD: 600 mg IV at weeks 0, 4, and 8	CD/UC: 180 or 360 mg SC q8w	11.5	Rescue dose: 1200 mg IV x1 Reduce dosing interval: q4, 6, 7 weeks
	UC: 1200 mg IV at weeks 0, 4, and 8			
Mirikizumab	CD: 900 mg IV at weeks 0, 4, 8	CD: 300 mg SC q4w	-	Extended induction: 600–1000 mg IV q4w for 3 doses
	UC: 300 mg IV at weeks 0, 4, 8	UC: 200 mg SC q4w		
Guselkumab	CD: 200 mg IV or 400 mg SC at weeks 0, 4, 8	CD/UC: 100 mg SC q8w or 200 mg SC q4w	-	-
	UC: 200 mg IV at weeks 0, 4, 8			
